# Intersectionality, vulnerability and foot health inequity

**DOI:** 10.1186/s13047-023-00647-7

**Published:** 2023-10-26

**Authors:** Joana Almeida, Jonathan Brocklehurst, Adrienne Sharples

**Affiliations:** 1 School of Applied Social Sciences, Faculty of Health and Social Sciences, University of Bedfordshire, Luton, UK; 2 Department of Podiatry, The SMAE Institute, Maidenhead, UK

**Keywords:** Foot health inequity, Social determinants, Intersectionality, Barriers, Vulnerability, UK

## Abstract

Foot health and wellbeing in the UK are often overlooked in healthcare. Foot health outcomes are strongly interlinked to the social determinants of health, in that the way these determinants intersect can impact an individual’s vulnerability to foot pain and disorders. In this commentary we explore some social determinants that hinder individuals from improving their foot health behaviour and ultimately reducing foot pain and foot disorder vulnerability. We focus on socioeconomic status, gender, disability, age, culture and ethnicity, and footwear quality; we also highlight the potential impact of the Covid-19 pandemic and the cost-of-living crisis on foot health inequities; rises in inflation have resulted in footcare becoming less affordable among vulnerable groups, like those with intellectual disabilities and chronic illness, older people, those living in rural and inner-city communities, and the ethnically and linguistically diverse population living in the UK. There is an urgent need to raise awareness of the social determinants of foot health, their intersectionality, and their impact on foot pain and disorder vulnerability. Despite the Black Report and both Marmot Reviews, little progress has been made in raising this awareness. It is recommended to widen the range of foot health interventions, by including it in GP consultations, developing cultural sensitivity within foot health services, creating more comprehensive educational foot health programmes, and developing a more sustainable footwear industry.

## Background

In the United Kingdom (UK), foot health and wellbeing have traditionally received less attention as a sub-area of healthcare. Moreover, the series of crises that the country has faced, from the Covid-19 pandemic to the cost-of-living crisis, the high inflation and the disruption of supply chains, may have had a significant impact on access to [foot] healthcare services across the UK for three key reasons [1]. Firstly, rising living costs have adversely affected the socioeconomic status of rural and inner-city communities, which have become further polarised from higher-income households. This has reduced disposable income in these communities, which has led to limited access to [foot] healthcare services, ultimately leading to higher instances of podiatric complaints [2, 3].

Secondly, the Covid-19 pandemic has resulted in a significant decrease in face-to-face healthcare services, with many healthcare providers adopting hybrid forms of communication with patients such as telemedicine [4]. Foot healthcare has benefited from this shift, as it has increased the potential reach of patient education. However, a main drawback has been a severe reduction in face-to-face treatment of low to moderate-risk foot pre-ulcerative lesions, such as Corns and Callus. A recent report by the Office for Health Improvement & Disparities highlighted that the Covid-19 pandemic had an impact on foot care, with a significant decrease in hospital admissions of diabetes patients between March 2020 and March 2021 and in major and minor amputations in March to June 2020 [5].

Thirdly, the significance of social determinants in achieving better patient outcomes has been highlighted by the Black Report and both Marmot Reviews [6–8]. According to the World Health Organization (WHO), ‘The social determinants of [foot] health (SDFH) are the non-medical factors that influence [foot] health outcomes [9]. They are the conditions in which people are born, grow, work, live, and age, and the wider set of forces and systems shaping the conditions of daily life. These forces and systems include economic policies and systems, development agendas, social norms, social policies, and political systems.’ Key social determinants outlined by the Marmot Reviews [7, 8], such as educational development, gender, ethnicity, life satisfaction and employment, highlight that a lack of attention to these determinants could create barriers to patient behavioural change, ultimately affecting foot health in the UK.

The intersectionality of the social determinants of foot health and foot health behaviours, as well as the most effective approach to prevent and challenge poor foot health habits among the general population but particularly among the most vulnerable, remains largely under-researched. Intersectionality refers to the overlap of different social determinants that contribute to our experiences of vulnerability or becoming vulnerable [10]. Someone’s degree of vulnerability therefore does not depend on one factor, but rather is multidimensional. Given this, we aim to comment on the barriers that have hindered adults from changing their foot health behaviors in the UK and worldwide, by using the social determinants of health lens and an intersectionality approach.

## Main text

Previous research [11] has demonstrated that foot health habits are strongly linked to other aspects of life and impact other areas of health and wellbeing. One example is the direct and indirect link between an individual’s socioeconomic status and their foot health. For patients deemed low or moderate risk of ulceration, private foot care is available, while public high-risk foot care is usually provided. This has resulted in the former group being forced to choose between financial burdens or neglecting their foot health. The latter group, meanwhile, has experienced increased uncertainty over appointment times when reliant on chronic wound management to prevent lower limb amputation. Furthermore, it is well-documented that those on a low income are more likely to develop type II diabetes and, once diagnosed, are more likely to develop foot related complications such as ulceration and amputation [5, 12–14]. Similarly, patients diagnosed with rheumatoid arthritis (RA), a condition with a high prevalence of foot pain and disorders complaints, and those belonging to high social deprivation categories struggle to access foot care and reported the impact of foot pain and disorders on the ability to work and on quality of life [15, 16].

In the UK, general practitioners (GPs) remain the primary point of contact for most NHS patients seeking assistance with foot health pain or disorders. Despite an increased demand for podiatric services [17], research has shown that patients often feel that their foot-related problems are overlooked or neglected by their GPs [18] and that their GPs focus on treating patients based on their condition, rather than their complaint [19]. Furthermore, the perception and limited understanding of the role of the podiatrist in healthcare and among patients, and the cuts in NHS services such as podiatry have further contributed to the limited public access and use of podiatry services [19], affecting most likely the most deprived segments of the UK population who heavily rely on NHS care and often lack the means to seek private healthcare.

In another study [20], foot pain, calluses, corns, nail pathologies, and structural deformities such as Hallux Abducto Valgus (HAV) were reported to be more commonly detected in females due to poor footwear habits, while fungal infections are more common in males. Consistent low socioeconomic position impacts on the ability to access new footwear and replace it when needed. Furthermore, the footwear industry has struggled to cater to the three-dimensional variation of feet in the population, leading the latter to wear functionally inadequate footwear. As an example, on one hand, less stock in shoe shops as half-sizes reduce the number of styles to be stocked; on the other hand, many shoe models are not available in half-sizes. Additionally, people do not buy footwear only to fit or only for comfort and mobility, but also based on style, colour, and occasion. A previous review of 18 international studies including the UK looking at correct shoe fit to foot shape, found that 63–72% of the population choose footwear that is a poor fit length/width/both [21]. The findings were strong in suggesting poorly fitting footwear results in foot pain, skin conditions (corns and callus), bony deformity such as HAV and lesser toe deformity, or ulceration if diabetic or with poor circulation. Other vulnerable groups also display a wider range of foot morphology; for example, those with intellectual disabilities like Down syndrome, older people, and those with diabetes are more likely to wear narrower footwear [21].

Finally, previous research has shown how podiatrists should ensure a culturally sensitive, patient-centred approach to managing high-risk podiatric clients from a refugee background, and ultimately from an ethnically and linguistically diverse population [22]. Some of their strategies include group education programs in languages other than English, client advocacy, working closely with family members and interpreters, negotiating health beliefs and customs and foot health behaviour changes, obtaining funding, and tackling social determinants that were impacting on foot health.

## Conclusion

Raising awareness about the social determinants of foot health, their intersectionality and their impact on foot pain and disorders susceptibility and vulnerability is an urgent need in the UK. Despite the Black Report from 43 years ago and the first Marmot Review 13 years ago, little progress has been made regarding this. In line with health inequities in general, more evidence is required to establish the link between the Covid-19 pandemic, the cost-of-living crisis, and widened foot health inequities. The outlook ahead to 2024 and beyond closely correlates with the trajectory of living costs, which suggests that as inflation rises and real wages remain stagnant, foot healthcare may become less affordable particularly among vulnerable groups in the UK. To address these issues, a range of interventions is recommended, such as demarginalising foot health and wellbeing among healthcare professionals (particularly GPs in primary care, who remain the gatekeepers of healthcare in the UK) and service users by proactively including it in regular GP consultations, developing cultural sensitivity in foot health services, creating a more sustainable footwear industry, developing educational foot health programmes to raise awareness of the importance of podiatry services, alongside more traditional interventions, such as good foot hygiene, good use of footwear, self-care, diet and lifestyle, and referrals.

## Data Availability

Not applicable.

## Abbreviations

GPs: General Practitioner(s)
HAV: Hallux Abducto Valgus
NHS: National Health Service
RA: Rheumatoid Arthritis
SDFH: Social Determinants of Foot Health
UK: United Kingdom
WHO: World Health Organization
